# Intraoperative performance and outcomes of robotic and laparoscopic total gastrectomy for gastric cancer: A high‐volume center retrospective propensity score matching study

**DOI:** 10.1002/cam4.5785

**Published:** 2023-03-16

**Authors:** Zhuoyu Jia, Shougen Cao, Cheng Meng, Xiaodong Liu, Zequn Li, Yulong Tian, Junjian Yu, Yuqi Sun, Jianfei Xu, Gan Liu, Xingqi Zhang, Hao Yang, Hao Zhong, Qingrui Wang, Yanbing Zhou

**Affiliations:** ^1^ Department of Gastrointestinal Surgery Affiliated Hospital of Qingdao University Qingdao China; ^2^ Shandong Provincial Key Laboratory of Gastrointestinal Tumor Basic and Translational Medicine Qingdao China

**Keywords:** gastric cancer, laparoscopic gastrectomy, long‐term survival, robotic gastrectomy

## Abstract

**Background:**

Studies on robotic total gastrectomy (RTG) are currently limited. This study aimed to compare the intraoperative performance as well as short‐ and long‐term outcomes of RTG and laparoscopic total gastrectomy (LTG).

**Methods:**

A total of 969 patients underwent robotic (*n* = 161) or laparoscopic (*n* = 636) total gastrectomy between October 2014 and October 2021. The two groups of patients were matched 1:3 using the propensity score matching (PSM) method. The intraoperative performance as well as short‐ and long‐term outcomes of the robotic (*n* = 147) and the laparoscopic (*n* = 371) groups were compared.

**Results:**

After matching, the estimated intraoperative blood loss was lower (80.51 ± 68.77 vs. 89.89 ± 66.12, *p* = 0.008), and the total number of lymph node dissections was higher (34.74 ± 12.44 vs. 29.83 ± 12.22, *p* < 0.001) in the RTG group compared with the LTG group. More lymph node dissections at the upper edge of the pancreas were performed in the RTG group than in the LTG (12.59 ± 4.18 vs. 10.33 ± 4.58, *p* = 0.001). Additionally, postoperative recovery indicators and laboratory data were greater in the RTG group than those in the LTG group, while postoperative complications were comparable between the two groups (19.0% vs. 18.9%, *p* = 0.962). For overweight or obese patients with body mass indexes (BMIs) ≥25, certain clinical outcomes of the RTG remained advantageous, and no significant differences in three‐year overall survival (OS) or relapse‐free survival (RFS) were observed.

**Conclusions:**

Robotic total gastrectomy demonstrated better intraoperative performance, could improve the short‐term clinical outcomes of patients, and was more conducive to patient recovery. However, the long‐term efficacies of the two approaches were similar. Robotic surgical systems may reduce surgical stress responses in patients, allowing them to receive postoperative chemotherapy sooner.

## Graphical abstract

Robotic total gastrectomy demonstrated better intraoperative performance, could improve the short‐term clinical outcomes of patients, and may reduce surgical stress responses in patients. The long‐term efficacies of the two approaches were similar.

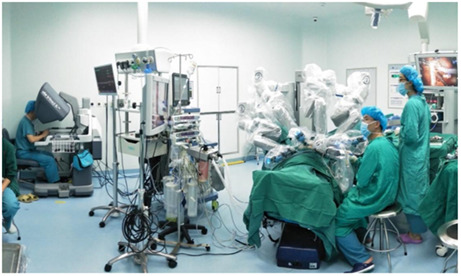

## 1 INTRODUCTION

Gastric cancer is the sixth most common and the third deadliest malignant tumor in the world.^1^ Since Kitano^2^ first reported the application of laparoscopic gastrectomy (LG) for gastric cancer (GC) in 1994, a growing body of studies has confirmed that in the treatment of gastric cancer, the efficacy of LG is comparable with open gastrectomy based on the short‐ and long‐term outcomes; in particular, the intraoperative performance of LG is better than that of open surgery.^3^, ^4^, ^5^, ^6^ However, laparoscopic surgery still has some inherent technical deficiencies, such as poor instrument mobility, poor visual field exposure, poor assistant coordination, and magnified hand tremors, leading to poor ergonomic surgical environments and a long learning curve.^7^, ^8^, ^9^ To overcome the limitations of laparoscopic surgery, a new generation of surgical robot systems, such as the da Vinci Robot, have been utilized. Through the technical advantages of three‐dimensional imaging, motion scaling, tremor filtering, 10‐fold amplification of the surgical visual field, increase in instrument freedom, and advanced ergonomic design, some technical shortcomings of laparoscopic surgery have been corrected.^9^ The use of a robotic system for the treatment of gastric cancer was first reported by Hashizume in 2002,^10^, ^11^ and robotic gastric surgery has since made great strides in reducing the learning curve^12^, ^13^, ^14^ and improving intraoperative performance,^15^, ^16^ enabling surgeons to perform more precise procedures than in laparoscopic surgeries. However, robotic surgeries for gastric cancer primarily focus on distal gastrectomy,^17^, ^18^, ^19^ and limited studies exist on their use in total gastrectomy procedures.^18^, ^20^, ^21^, ^22^

Minimally invasive total gastrectomy is one of the most technically demanding operations in gastric cancer surgery and includes precise, extensive lymph node dissection and high‐quality digestive tract reconstruction according to Japanese gastric cancer treatment guidelines. D2 lymph node dissection, using Roux‐en‐Y digestive tract reconstruction, is suitable for all advanced patients, including in nodes No. 1–7, No. 8a, 9, 11p, 11d, and 12a.^23^ Intracorporeal mechanically stapled Roux‐en‐Y anastomosis is a key step and challenge in the process of digestive tract reconstruction, particularly in patients with obesity.^24^ Few studies exist on whether the robotic surgery system has technical advantages or whether it can improve the surgical results and long‐term survival of total gastrectomy patients,^20^, ^21^, ^22^ and controversy regarding which gastric cancer method is superior may exist, some umbrella reviews or metanalyses of metanalyses have shown that the safety and effectiveness of robotic gastrectomy still need strong evidence to prove that a larger sample size and clinical trials are needed to support the advantages of robotic surgery.^25^, ^26^, ^27^ We performed the first DaVinci robotic surgical system‐assisted radical gastrectomy on October 14, 2014, and have since performed more than 1000 of these procedures.

In this retrospective study, we used propensity score matching (PSM) analysis to compare the differences between robotic and laparoscopic total gastrectomy in intraoperative performance as well as short‐ and long‐term outcomes. Focus on the characteristics of robot surgery system in total gastrectomy, explore its safety and effectiveness, and analyze the long‐term oncology results. In addition, further study the advantages of robot surgery for overweight and obese patients. We aimed to investigate whether some underlying mechanisms of robotic total gastrectomy may reduce the surgical stress response and improve tumor prognosis for gastric cancer patients.

## 2 METHODS

### 2.1 Design and patients

**FIGURE 1:**
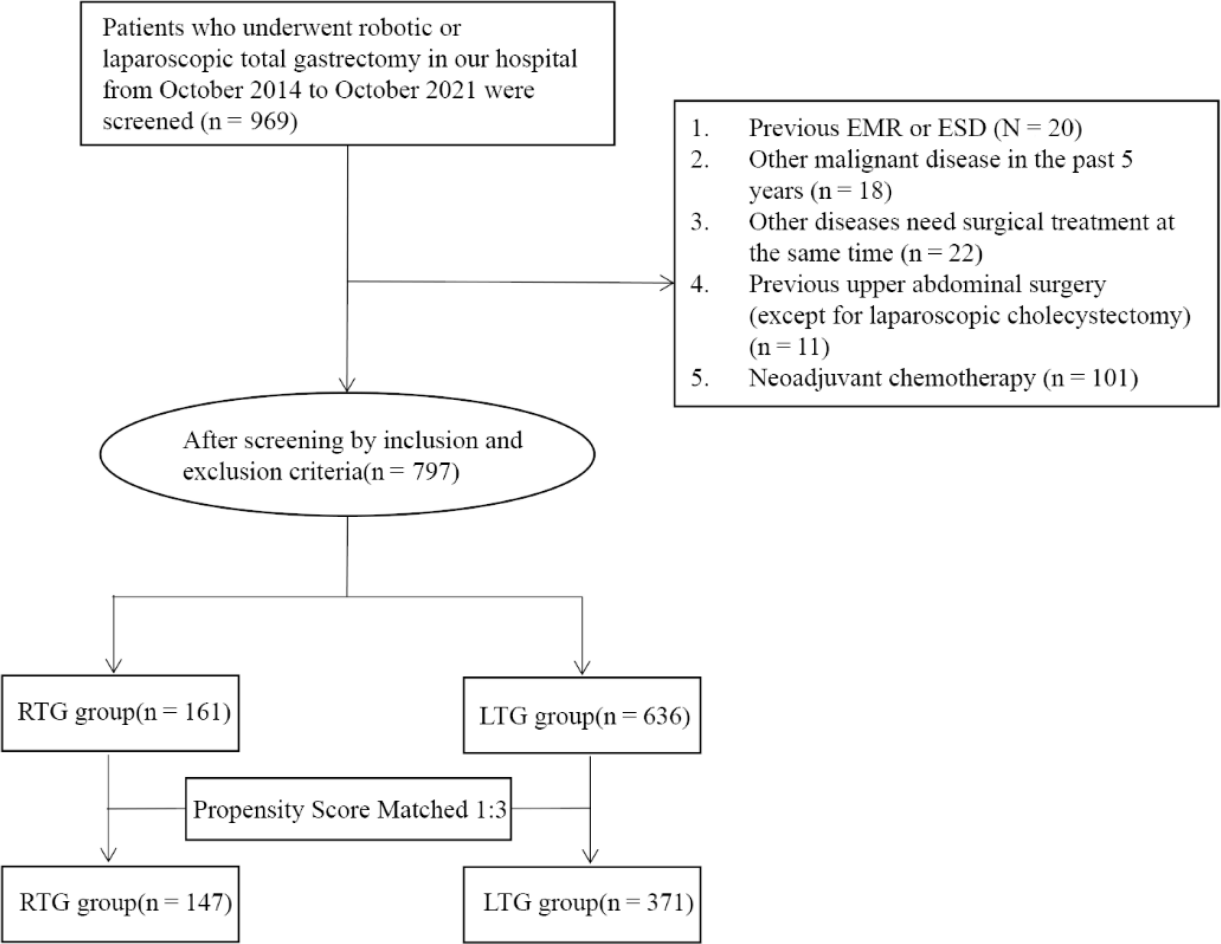
Flowchart of patients enrolled in this study. EMR, endoscopic mucosal resection; ESD, endoscopic submucosal dissection; RTG, robotic total gastrectomy; LTG, laparoscopic total gastrectomy.

This was a retrospective study and was reviewed by the appropriate ethics committee, ethics number: QYFY WZLL 27151, all patients signed informed consent. The inclusion criteria included patients who underwent robotic or laparoscopic total gastrectomy at our center from October 2014 to October 2021; aged 18–75 years; diagnosed with gastric adenocarcinoma by gastroscopic biopsy; had cT2‐4aN0/+M0 (patient staging was adjusted according to the 8th edition of the American Joint Commission on Cancer (AJCC) staging system^28^); had a tumor located in the middle or upper part of the stomach; classified as American Society of Anesthesiologists (ASA) ≤3; and had Karnofsky Performance Scale (KPS) scores ≥60. The exclusion criteria included patients undergoing neoadjuvant therapy; with esophagogastric junction cancer; who had previously undergone gastrectomy, endoscopic mucosal resection, or endoscopic submucosal dissection; other malignant diseases in the previous 5 years; heart, lung, liver, and kidney insufficiency or a history of cerebral infarction; emergency surgery for complications of gastric cancer (bleeding, obstruction, or perforation); other diseases requiring simultaneous surgery; and previous upper abdominal surgery (with the exception of laparoscopic cholecystectomy). In addition, this study analyzed overweight or obese patients with BMIs ≥25 and examined the intraoperative performance and clinical outcomes of such patients in the two groups. The design of this study is shown in Figure 1.

The surgical method, either laparoscopic gastrectomy (LG) or robotic gastrectomy (RG), was decided by each patient. Both laparoscopic and robotic surgeries were performed by the same surgical team, and all surgeons had performed either more than 100 laparoscopic total gastrectomy (LTG) or 100 robotic total gastrectomy (RTG) surgeries.^29^

As this was a retrospective study, patients were screened according to rigorous inclusion and exclusion criteria. To eliminate confounding factors and resolve possible patient selection biases, the following variables were used for PSM: age, sex, BMI, ASA grade, Nutrition Risk Screening 2002 (NRS‐2002 score), tumor size, tumor location, pathological stage, and tumor differentiation. We performed 1:3 matching using a 0.2 caliper width. Ultimately, 147 and 371 patients were included in the RTG and LTG groups, respectively. After matching, the baseline data of the two groups were balanced.

### 2.2 Perioperative management and surgical procedure

All patients in this study underwent the perioperative Enhanced Recovery after Surgery (ERAS) protocol in our center,^30^, ^31^, ^32^, ^33^ and no differences between the two groups were noted. The robotic surgeries utilized the Da Vinci® Si™ Surgical System, and all patients underwent standard radical total gastrectomy and D2 lymph node dissection according to the Japanese gastric cancer treatment guidelines.^23^ Both groups underwent computerized tomography angiography (CTA) to determine the anatomical features and variations of the perigastric artery prior to operations and to inform preoperative operation decisions by the multidisciplinary team (MDT).^34^ No significant difference in the surgical steps existed between the two groups. The LTG group used a wire to suspend the liver to expose the surgical field, and the RTG group used the No. 3 robotic arm to expose the field of view, reducing additional damage.

All patients received intracorporeal mechanically stapled Roux‐en‐Y anastomosis for gastrointestinal reconstruction combined with π‐shaped esophagojejunostomy or overlap anastomosis after total gastrectomy, as previously described.^35^, ^36^, ^37^

According to the postoperative pathological and molecular detection of patients, it is recommended that patients with high‐risk stages II and above adopt adjuvant chemotherapy with a 5‐fluorouracil‐based chemotherapy regimen for 6–8 cycles.^23^ Follow‐up was performed every 3–6 months for 3 years, every 6 months for 3–5 years, and every year after 5 years. Follow‐up items included routine blood tests, blood biochemistry, tumor markers, abdominal computed tomography (CT), and electronic gastroscopy.^23^ We reviewed postoperative outpatient medical records and conducted telephone or WeChat interviews for patients who had not completed follow‐ups. Follow‐ups were conducted through May 2022. All of the matched patients completed the follow‐ups, and the survival status of patients was noted.

### 2.3 Data collection

Clinical data of patients were collected prospectively in our center's database and retrospectively through patient medical records. The following data were obtained: patient characteristics (age, sex, and BMI), preoperative physical status (ASA physical status), nutritional status (NRS‐2002 scores^38^), laboratory data (various hematological preoperative and postoperative test results), operation status (including operation time, intraoperative blood loss, intraoperative blood transfusion, and conversion to laparotomy), postoperative pathological report (including number of lymph nodes dissected, number of lymph node metastases, tumor size, tumor location, pathological stage, and tumor differentiation degree), and postoperative recovery (including postoperative drainage volume, postoperative bowel function recovery time (exhaust or defecate) and first liquid diet, postoperative hospital stay, postoperative complications, and hospitalization costs). Complications were classified according to the Clavien–Dindo (CD)^39^, ^40^ classification, and statistics were calculated based on the higher CD grade in patients with more than one complication. Finally, long‐term survival was determined based on outpatient revisits or telephone follow‐ups.

### 2.4 Statistical analyses

The normality of the distribution of continuous variables was assessed using the Kolmogorov–Smirnov method. Quantitative data with normal distributions were compared using t‐tests, while those with non‐normal distributions were compared using the Mann–Whitney U test. Categorical data are expressed using frequencies and percentages, and were compared using χ^2^ or Fisher's exact tests. The Mann–Whitney U test was used to compare ordinal data between groups. Survival curves were plotted using the Kaplan–Meier method, and the survival rate was calculated. The log‐rank test was used to compare differences between the two groups. All analyses were performed using IBM SPSS Statistics 24 (SPSS), with *p* < 0.05 (two‐tailed *p*‐value) indicating statistical significance.

## 3 RESULTS

### 3.1 Basic characteristics

The annual number of surgical cases of LTG and RTG between 2014 and 2021 is shown in Figure 3B, which also shows evidence that the number of robotic total gastric surgeries has increased in recent years. Additionally, basic characteristics of the two groups before and after PSM are shown in Table 1. Overall, 797 patients were included in this study, with 161 and 636 patients in the RTG and LTG groups, respectively. Significant differences were observed in sex, BMI, ASA grade, and pT stage between the two groups prior to matching (*p* < 0.05). After PSM at a ratio of 1:3, 147 and 371 patients were included in the RTG and LTG groups, respectively. The baseline data of the two groups were well‐balanced.

**TABLE 1:** Basic characteristics of patients in RTG and LTG groups before and after propensity score matching.

Variable	Before matching			After matching		
	RTG (*n* = 161)	LTG (*n* = 636)	*p*	RTG (*n* = 147)	LTG (*n* = 371)	*p*
	Mean ± SD/N	Mean ± SD/N		Mean ± SD/N	Mean ± SD/N	
Age, years	63.35 ± 9.85	61.92 ± 9.26	0.153	62.92 ± 10.00	62.52 ± 9.43	0.927
Sex						
Male	132 (82.0%)	474 (74.5%)	0.048*	118 (80.3%)	294 (79.2%)	0.794
Female	29 (18.0%)	162 (25.5%)		29 (19.7%)	77 (20.8%)	
BMI, kg/m^2^	25.14 ± 3.72	24.23 ± 3.25	0.005*	24.97 ± 3.70	24.54 ± 3.35	0.249
ASA, score						
1–2	111 (68.9%)	338 (53.1%)	<0.001*	97 (66.0%)	233 (62.8%)	0.497
3	50 (31.1%)	298 (46.9%)		50 (34.0%)	138 (37.2%)	
Comorbidity						
Hypertension	30 (18.6%)	162 (25.5%)	0.070	22 (15.0%)	62 (16.7%)	0.627
Diabetes	33 (20.5%)	140 (22.0%)	0.677	29 (19.7%)	74 (19.9%)	0.955
Coronary	17 (10.6%)	79 (12.4%)	0.517	13 (8.8%)	37 (10.0%)	0.695
NRS2002						
<3	63 (39.1%)	274 (43.1%)	0.365	61 (41.5%)	167 (45.0%)	0.467
≥3	98 (60.9%)	362 (56.9%)		86 (58.5%)	204 (55.0%)	
Size, cm	4.99 ± 2.63	5.28 ± 2.88	0.293	5.07 ± 2.69	5.03 ± 2.80	0.800
Tumor location						
U	99 (61.5%)	416 (65.4%)	0.353	90 (61.2%)	239 (64.4%)	0.496
M	62 (38.5%)	220 (34.6%)		57 (38.8%)	132 (35.6%)	
pT^a^						
pT1	29 (18.0%)	83 (13.1%)	0.012*	29 (19.7%)	63 (17.0%)	0.898
pT2	45 (28.0%)	139 (21.9%)		39 (26.5%)	101 (27.2%)	
pT3	58 (36.0%)	321 (50.5%)		57 (38.8%)	152 (41.0%)	
pT4a	29 (18.0%)	93 (14.6%)		22 (15.0%)	55 (14.8%)	
pN^a^						
pN0	68 (42.2%)	238 (37.4%)	0.665	62 (42.2%)	150 (40.4%)	0.521
pN1	33 (20.5%)	143 (22.5%)		28 (19.0%)	77 (20.8%)	
pN2	23 (14.3%)	108 (17.0%)		21 (14.3%)	69 (18.6%)	
pN3	37 (23.0%)	147 (23.1%)		36 (24.5%)	75 (20.2%0	
AJCC8th^a^						
I	44 (27.3%)	177 (27.8%)	0.992	44 (29.9%)	121 (32.6%)	0.728
II	48 (29.8%)	189 (29.7%)		41 (27.9%)	92 (24.8%)	
III	69 (42.9%)	270 (42.5%)		62 (42.2%)	158 (42.6%)	
Histologic type						
Well/moderately	115 (71.4%)	428 (67.3%)	0.315	105 (71.4%)	270 (72.8%)	0.757
Poorly/undifferentiated	46 (28.6%)	208 (32.7%)		42 (28.6%)	101 (27.2%)	

Abbreviations: ASA, American Society of Anesthesiologists; BMI, body mass index; NRS, nutrition risk screening.

* Statistically significant.

^a^ Pathologic stage according to the American Joint Committee on Cancer, 8th Edition.

### 3.2 Surgical outcomes

Intraoperative performance is shown in Table 2. The total operation time refers to the time from skin incision to closing the incision, including the actual operation time (operation steps under the robot or laparoscopy) and auxiliary operation time (including establishment of the trocar, docking and withdrawal of instrument arms, extraction of specimens through auxiliary small incisions, examination of surgical areas, and placement of drainage tubes). A statistically significant difference was noted in the total operation time (279.82 ± 60.64 vs. 264.01 ± 72.43, *p* = 0.008), while the actual operation time (200.61 ± 60.19 vs. 206.52 ± 72.52, *p* = 0.385) showed no significant difference. Thus, a difference in operation time between the RTG and LTG groups was observed in the auxiliary operation time (73.44 ± 11.27 vs. 69.27 ± 13.04, *p* < 0.001). The estimated intraoperative blood loss in the RTG group was lower than that in the LTG group (80.51 ± 68.77 vs. 89.89 ± 66.12, *p* = 0.008), and no significant differences were noted in intraoperative blood transfusion, positive margin rate, or conversion to laparotomy between the two groups (*p* > 0.05).

**TABLE 2:** Surgical outcomes, postoperative recovery, and postoperative complications in the RTG and LTG groups after propensity score matching.

Variables	After matching		*p*
	RTG (*n* = 147)	LTG (*n* = 371)	
	Mean ± SD/*N*(%)	Mean ± SD/*N*(%)	
Surgical outcomes			
Total operative time (min)	279.82 ± 60.64	264.01 ± 72.43	0.008*
The robot/laparoscopy time (min)	200.61 ± 60.19	206.52 ± 72.52	0.385
The assisted time (min)	73.44 ± 11.27	69.27 ± 13.04	<0.001*
Estimated blood loss (mL)	80.51 ± 68.77	89.89 ± 66.12	0.008*
Total examined LNs	34.74 ± 12.44	29.83 ± 12.22	<0.001*
Examined suprapancreatic LN	12.56 ± 4.25	10.74 ± 4.53	<0.001*
Total metastatic LNs	4.64 ± 8.21	3.96 ± 6.39	0.932
Intraoperative transfusion	3 (2.0%)	4 (1.1%)	0.665
Positive resection margin	0 (0%)	0 (0%)	—
Open conversion	0 (0%)	8 (2.2%)	0.162
Postoperative recovery			
Amylase in drainage fluid (U/L)	315.29 ± 316.77	435.44 ± 404.33	<0.001*
Drainage on the first day after operation (ml)	108.83 ± 56.52	112.77 ± 54.05	0.517
Bowel function recovery (days)	3.22 ± 0.62	3.45 ± 0.58	<0.001*
First liquid diet after surgery (days)	3.70 ± 0.60	3.95 ± 0.73	<0.001*
Postoperative hospital stays (days)	10.25 ± 5.45	11.30 ± 6.96	0.036*
Postoperative chemotherapy interval (days)*	28.88 ± 7.45	31.76 ± 8.30	0.002*
Postoperative mortality	0 (0%)	0 (0%)	—
Unplanned reoperation	2 (1.4%)	7 (1.9%)	0.968
Unplanned readmission	5 (3.4%)	10 (2.7%)	0.888
Medical cost ($)	18104.32 ± 5476.96	14095.19 ± 7277.92	0.001*
Postoperative complications	28 (19.0%)	70 (18.9%)	0.962
Anastomotic leakage	3 (2.0%)	8 (2.2%)	1.000
Pulmonary	9 (6.1%)	26 (7.0%)	0.717
Abdominal infection	1 (0.7%)	6 (1.6%)	0.681
Intra‐abdominal bleeding	2 (1.4%)	8 (2.2%)	0.811
Gastrointestinal bleeding	2 (1.4%)	2 (0.5%)	0.320
Pleural effusion	5 (3.4%)	14 (3.8%)	0.839
Lymphatic leakage	0 (0%)	2 (0.5%)	1.000
Pancreatic leakage	0 (0%)	3 (0.8%)	0.562
Wound problem	3 (2.0%)	5 (1.3%)	0.856
Ileus	5 (3.4%)	10 (2.7%)	0.888
Clavien–Dindo classification			
0	119 (81.8%)	301 (81.1%)	0.937
I–II	21 (14.3%)	50 (13.5%)	
III–IV	7 (4.8%)	20 (5.4)	
V	0 (0%)	0 (0%)	

Abbreviations: LN, lymph node; LTG, laparoscopic total gastrectomy; RTG, robotic total gastrectomy.

* Statistically significant.

Moreover, a statistically significant difference in the total number of lymph nodes dissected between the RTG and LTG groups was observed (34.74 ± 12.44 vs. 29.83 ± 12.22, *p* < 0.001). Notably, the number of lymph nodes dissected in the upper border of the pancreas was also significantly different between the two groups (12.56 ± 4.25 vs. 10.74 ± 4.53, *p* < 0.001). No significant differences in the positive rate of dissected lymph nodes between the two groups were noted (4.64 ± 8.21 vs. 3.96 ± 6.39, *p* = 0.932).

### 3.3 Postoperative short‐term outcomes

Postoperative recovery outcomes are shown in Table 2. The time of bowel function recovery (3.70 ± 0.60 vs. 3.95 ± 0.73 days, *p* < 0.001), first liquid diet intake (3.70 ± 0.60 vs. 3.95 ± 0.73 days, *p* < 0.001), postoperative hospital stays lengths (10.25 ± 5.45 vs. 11.30 ± 6.96 days, *p* = 0.036), and postoperative adjuvant chemotherapy intervals (28.88 ± 7.45 vs. 31.76 ± 8.30 days, *p* = 0.002) were significantly shorter in the RTG group than in the LTG group. No significant differences were noted in the abdominal drainage volume on the first day after surgery (108.83 ± 56.52 vs. 112.77 ± 54.05 mL), unplanned reoperation rate, or unplanned readmission rate between the two groups (*p* > 0.05). No postoperative deaths occurred in either group, and the total cost of hospitalization for the RTG group was significantly higher than that for the LTG group (18104.32 ± 5476.96 vs. 14095.19 ± 7277.92 dollars, *p* = 0.001).

Postoperative complications are shown in Table 2, which shows that the overall rates of complications in the two groups were similar (19.0% vs. 18.9%, *p* = 0.962). This study further compared the incidence of specific complications, such as anastomotic fistula, pneumonia, abdominal infection, intra‐abdominal bleeding, gastrointestinal bleeding, pleural effusion, lymphatic fistula, pancreatic fistula, incision problems (including incision dehiscence, incision infection, or need for open incision treatment), and intestinal obstruction. No significant differences in the occurrence of these complications were observed between the two groups (*p* > 0.05).

### 3.4 Laboratory data

**FIGURE 2:**
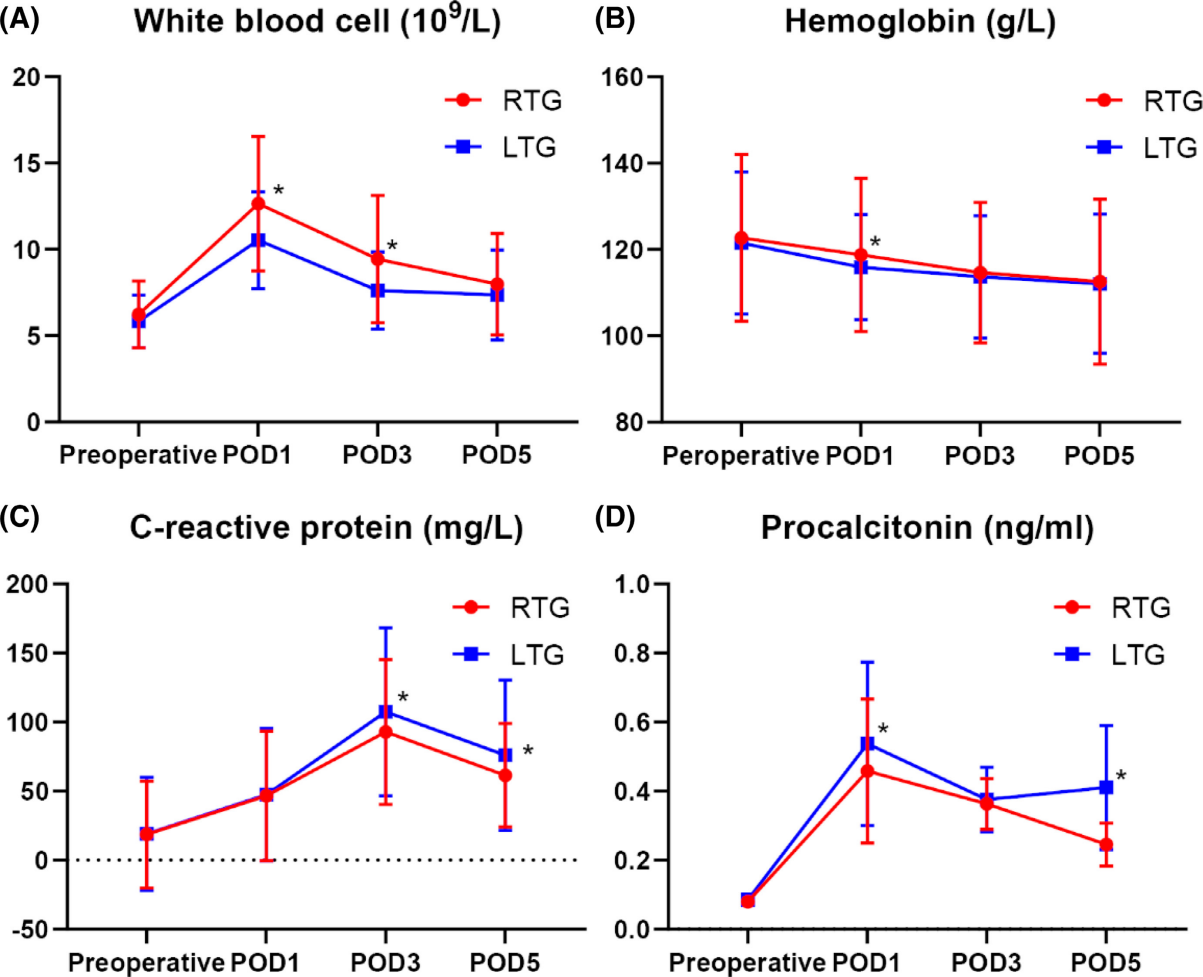
Laboratory data, white blood cells (A), hemoglobin (B), C‐reactive protein (C), procalcitonin (D). *Statistically significant.

**FIGURE 3:**
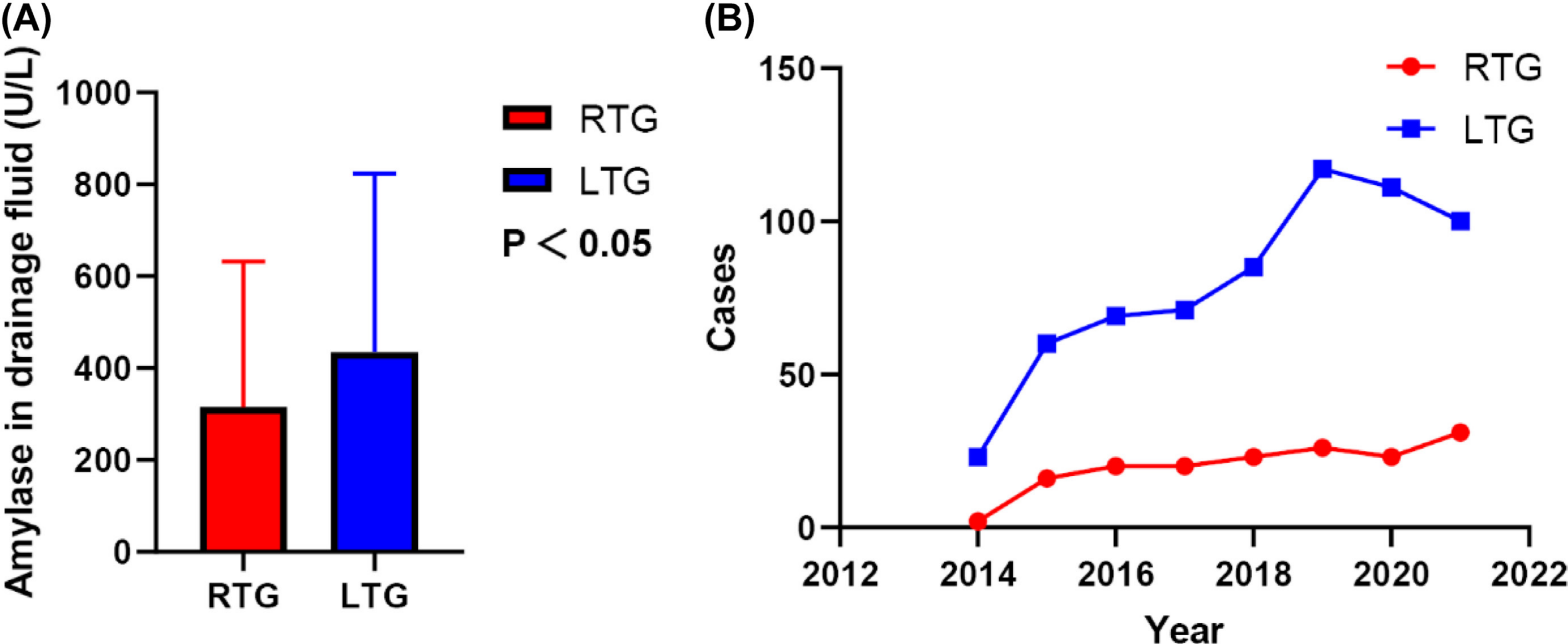
Drainage fluid amylase (A), and the annual number of surgical cases of two groups from 2014 to 2021 (B).

Figure 2 shows the laboratory data of the two groups. In addition to testing the relevant indicators before surgery, laboratory indicators from both groups were obtained on the first‐, third‐, and fifth‐days of postoperation (POD1, POD3, and POD5), including white blood cells (WBC), hemoglobin (HB), C‐reactive protein (CRP), procalcitonin (PCT), and drainage fluid amylase (Figure 3A), which were compared in each group. There were significant differences in POD1WBC, POD3WBC, POD1HB, POD3CRP, POD5CRP, POD1PCT, and POD5PCT between the two groups (*p* < 0.05). Notably, the level of amylase in POD1 drainage fluid (315.29 ± 316.77 vs. 435.44 ± 404.33, *p* < 0.05) was significantly lower in the RTG group than in the LTG group.

### 3.5 Data of the two groups of patients with BMI ≥25 after matching

The data of patients with BMIs ≥25 in the two groups after matching are shown in Table 3. Similar to the overall cohort after matching, among patients with BMIs ≥25, the RTG and LTG groups showed significant differences in total operation time (289.45 ± 65.58 vs. 271.14 ± 64.77 min, *p* = 0.075), adjuvant operation time (72.58 ± 10.11 vs. 69.23 ± 12.84 min, *p* = 0.001), estimated intraoperative blood loss (76.86 ± 64.51 vs. 88.08 ± 64.17 mL, *p* = 0.04), total number of lymph node dissections (33.40 ± 9.84 vs. 28.47 ± 11.34, *p* < 0.001), number of lymph node dissections in the upper border of the pancreas (12.59 ± 4.18 vs. 10.33 ± 4.58, *p* = 0.001), recovery time of bowel function (3.43 ± 0.52 vs. 3.63 ± 0.63 days, *p* = 0.007), time of first liquid diet after operation (3.82 ± 0.62 vs. 4.17 ± 0.77 days, *p* < 0.001), interval of starting adjuvant chemotherapy after the operation (28.62 ± 7.27 vs. 32.04 ± 8.23 days, *p* = 0.004), and total medical expenses (18977.58 ± 6939.88 vs. 14095.19 ± 6332.04$, *p* < 0.001). Of note, the procedure for eight patients in the overall cohort was converted to laparotomy, of whom five patients were overweight and all were in the LTG group. Of these, the procedure for one patient was converted to laparotomy due to intraoperative hemorrhage, two due to tumor sites, and two due to obesity. No significant differences were observed between the two groups in terms of postoperative complications (*p* > 0.05).

**TABLE 3:** Surgical outcomes, postoperative recovery, and postoperative complication in the RTG and LTG groups after propensity score matching.

Variables	After matching		*p*
	RTG (*n* = 78)	LTG (*n* = 167)	
	Mean ± SD/*N*(%)	Mean ± SD/*N*(%)	
Surgical outcomes			
Total operative time (min)	289.45 ± 65.58	271.14 ± 64.77	0.075
The robot/laparoscopy time (min)	210.26 ± 65.21	213.79 ± 64.75	0.513
The assisted time (min)	72.58 ± 10.11	69.23 ± 12.84	0.001*
Estimated blood loss (mL)	76.86 ± 64.51	88.08 ± 64.17	0.040*
Total examined LNs	33.40 ± 9.84	28.47 ± 11.34	<0.001*
Examined suprapancreatic LN	12.59 ± 4.18	10.33 ± 4.58	0.001*
Total metastatic LNs	4.06 ± 5.85	3.99 ± 6.44	0.832
Intraoperative transfusion	1 (1.3%)	1 (0.6%)	0.536
Open conversion	0 (0%)	5 (3.0%)	0.181
Postoperative recovery			
Amylase in drainage fluid (U/L)	355.08 ± 330.03	430.53 ± 348.03	0.025*
Drainage on the first day after operation (ml)	110.55 ± 56.18	115.39 ± 56.24	0.548
Bowel function recovery (days)	3.43 ± 0.52	3.63 ± 0.63	0.007*
First liquid diet after surgery (days)	3.82 ± 0.62	4.17 ± 0.77	<0.001*
Postoperative hospital stays (days)	11.13 ± 5.44	11.82 ± 7.66	0.675
Postoperative chemotherapy interval (days)*	28.62 ± 7.27	32.04 ± 8.23	0.004*
Unplanned reoperation	2 (2.6%)	5 (3.0%)	1.000
Unplanned readmission	3 (3.8%)	7 (4.2%)	1.000
Medical cost ($)	18977.58 ± 6939.88	14095.19 ± 6332.04	<0.001*
Postoperative complication	17 (21.8%)	32 (19.2%)	0.631
Anastomotic leakage	3 (3.8%)	5 (3.0%)	1.000
Pulmonary	4 (5.1%)	11 (6.6%)	0.875
Abdominal infection	1 (1.3%)	4 (2.4%)	1.000
Intra‐abdominal bleeding	2 (2.6%)	4 (2.4%)	1.000
Gastrointestinal bleeding	1 (1.3%)	1 (0.6%)	0.536
Pleural effusion	4 (5.1%)	8 (4.8%)	1.000
Lymphatic leakage	0 (0%)	1 (0.6%)	1.000
Pancreatic leakage	0 (0%)	2 (1.2%)	1.000
Wound problem	1 (1.3%)	5 (3.0%)	0.668
Ileus	2 (2.6%)	4 (2.4%)	1.000
Clavien–Dindo classification			
0	61 (78.2%)	135 (80.8%)	0.880
I–II	11 (14.1%)	20 (12.0%)	
III–IV	6 (7.7%)	12 (7.2%)	
V	0 (0%)	0 (0%)	

Abbreviations: LN, lymph node; LTG, laparoscopic total gastrectomy; RTG, robotic total gastrectomy.

* Statistically significant.

### 3.6 Long‐term oncology results

#### 3.6.1 Comparison by tumor pathological stage

**FIGURE 4:**
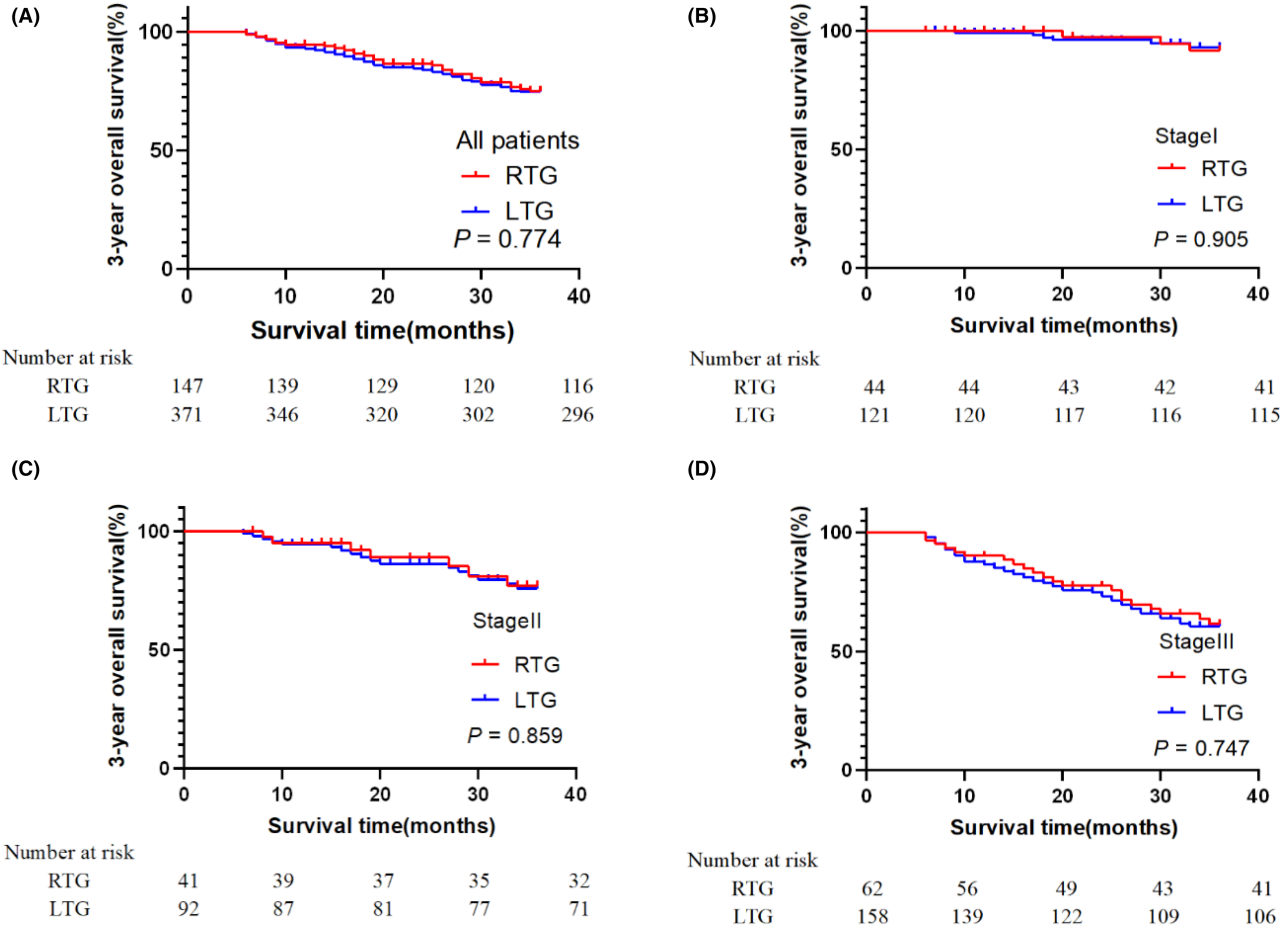
Kaplan–Meier estimates of overall survival of all patients (A), patients with stage I disease (B), patients with stage II disease (C), and patients with stage III disease (D).

The three‐year overall survival rate (OS) and relapse‐free survival (RFS) rate of the two groups were examined. The three‐year OS rates of the RTG and LTG groups were 78.9% and 79.8%, respectively. Kaplan–Meier analyses of three‐year OS showed that the difference between the two groups was not statistically significant (log‐rank, *p* = 0.774, Figure 4A). The overall survival rate of the two groups was also analyzed according to the pathological stage, of which all patients had stage I (93.2% RTG group vs. 95.0% LTG group, *p* = 0.905, Figure 4B), stage II (82.9% RTG group vs. 81.5% LTG group, *p* = 0.859, Figure 4C), or stage III (66.1% RTG group vs. 67.1% LTG group, *p* = 0.747, Figure 4D). Stage analyses showed that there was no significant difference in the three‐year OS between the two groups in stages I, II, or III.

During the follow‐up period, some patients experienced tumor recurrence, and the three‐year RFS rate was compared between the two groups. The rates of the RTG and LTG groups were 74.8% and 76.0%, respectively. The Kaplan–Meier analyses of RFS showed no statistically significant differences between the two groups (log‐rank, *p* = 0.656, Figure S5). The three‐year RFS in stage I (90.9% RTG group vs. 92.6% LTG group, *p* = 0.930, Figure S5), stage II (78.0% RTG group vs. 77.2% LTG group, *p* = 0.916, Figure S5), and stage III patients (61.3% RTG group vs. 62.7% LTG group, *p* = 0.621, Figure S5) was calculated, which also showed no statistically significant differences.

#### 3.6.2 Comparison by lymph node status

The long‐term oncology results of the two groups are shown in Figure S6. According to whether the lymph node metastasis was positive, the three‐year OS and RFS of the RTG and LTG groups were compared. The three‐year OS of pN0 patients was 88.7% and 88.0% in the RTG group and LTG groups, respectively, and Kaplan–Meier analyses showed that the difference between the two groups was not statistically significant (log‐rank, *p* = 0.683, Figure S6A). Additionally, the three‐year RFS in pN0 patients was 85.5% and 85.3% in the RTG and LTG groups, respectively (*p* = 0.536, Figure S6B). For pN+ patients, the three‐year OS was 71.8% and 73.8% in the RTG and LTG groups, respectively (*p* = 0.942, Figure S6C), while the three‐year RFS was 67.1% in the RTG group and 69.1% in the LTG group (*p* = 0.972, Figure S6D). No significant differences were observed in the three‐year OS or RFS performance between the RTG and LTG groups, regardless of whether they were pN0 or pN+.

#### 3.6.3 Comparison by BMI ≥25

The long‐term oncology results of the two groups are shown in Figure S6. For patients with BMIs ≥25, the three‐year OS was 84.6% and 77.8% in the RTG and LTG groups, respectively. Kaplan–Meier analyses showed that the difference between the two groups was not statistically significant (log‐rank, *p* = 0.178, Figure S6E). The three‐year RFS in overweight or obese patients was 80.8% and 75.4% in the RTG and LTG groups, respectively (*p* = 0.256, Figure S6F). Similarly, however, no significant differences were noted between the two groups.

## 4 DISCUSSION

In this study, PSM analyses showed that the robotic surgical system showed equivalent performances to laparoscopic surgery in terms of intraoperative performance, short‐term clinical outcomes, and long‐term survival. The procedure appeared to be a stable, reliable, and promising minimally invasive surgical method, particularly in some overweight patients. Although no significant differences were seen in overall complication rates, three‐year OS, and RFS between the two groups, compared with LTG, RTG was associated with less intraoperative bleeding, less trauma, fewer inflammatory reactions, more lymph node retrieval, and faster postoperative recovery. Therefore, it may be reasonable to speculate that the RTG group had better clinical outcomes.

The surgical safety of LG in the treatment of gastric cancer has been proven through a number of large randomized clinical trials.^3^, ^4^, ^5^ With the development of minimally invasive surgeries, patients may be more inclined to choose laparoscopic and robotic procedures, which have become more popular due to these advancements. In our retrospective study with PSM, compared with LTG, RTG showed clear advantages in surgical results, postoperative recovery, and inflammatory indicators. The system has the advantages of clearer three‐dimensional operating fields and imaging, motion zoom, and tremor filtering, and the intraoperative blood loss was significantly lower than that of laparoscopic surgery.^41^, ^42^ In terms of operation time, although the total operation time in the RTG group was longer, there were no significant differences in the actual operation time (time of operation steps under the robot or laparoscopy) between the two groups, with the differences between the two groups being caused by secondary auxiliary operation time (including the establishment of trocar, docking and withdrawal of instrument arms, extraction of specimens through auxiliary small incisions, examination of surgical areas, and placement of drainage tubes). Moreover, the number of lymph nodes obtained as an index to evaluate the quality of operation is related to the accurate evaluation of pathological stage, prognosis, and follow‐up treatment^43^, ^44^, ^45^ and is beneficial to the survival of patients with gastric cancer.^46^, ^47^ The total number of lymph nodes dissected in the RTG group was greater than that in the LTG group. The robot helped to improve the stability, efficiency, and accuracy of the operation. Consequently, the RTG group could obtain more precise dissection, particularly of those in the upper border of the pancreas. Additionally, the number of lymph nodes was greater, and the value of amylase in the postoperative drainage fluid was lower, indicating that robotic surgeries were more precise in the dissection of the upper border region of the pancreas and caused less trauma to the pancreas. Robotic surgery may also effectively avoid excessive compression of the pancreas during surgical procedures.^19^, ^48^ Multicenter studies based on different surgical experiences have shown that mild intraoperative events may lead to higher postoperative complications and mortality.^49^, ^50^ The difference in inflammatory indices on the first‐, third‐, and fifth‐day postoperation also reflected that the RTG group had less surgical trauma and a lower inflammatory response, which resulted in faster postoperative recovery. Although the interval of adjuvant chemotherapy in the RTG group is shorter than that in the LTG group, it still needs to be confirmed by a large sample of high‐quality randomized controlled trials. No significant differences were observed between the two groups in terms of the incidence of complications. However, patients in the RTG group recovered faster in postoperative bowel function, spent less time on a liquid diet, had shorter postoperative hospital stays, all of which were more beneficial to the recovery of patients. Robotic surgeries are also advantageous for short‐term clinical outcomes.^18^, ^21^, ^22^, ^51^, ^52^ After Japan incorporated robotic gastrectomy into the universal health insurance coverage, the safety of robotic surgery was further confirmed.^53^ However, robotic surgery is not included in the universal health insurance coverage in China, and the medical cost of the RTG group was more expensive, which may limit the widespread application of robotic surgery systems. Additionally, multicenter, large sample, high‐quality randomized control trials (RCT) to confirm these findings in total gastrectomy cases are still warranted.

This study analyzed overweight or obese patients with BMIs ≥25. The clinical outcomes of these patients were similar to those of the matched overall cohort, and the RTG group had advantages in terms of surgical results and postoperative recovery. Notably, the procedure for eight patients in the overall cohort was converted to laparotomy, of whom five patients were overweight and all were in the LTG group. Robotic surgery had significantly better visual field exposure than laparoscopic surgery in overweight patients. For overweight or obese patients, RTG may be a better surgical method when choosing minimally invasive surgery among various possible methods.

Due to recent positive clinical outcomes of RTG, the number of robotic surgeries has increased. However, research on whether RTG can improve long‐term survival remains limited. In this study, three‐year OS and three‐year RFS were observed in the RTG and LTG groups according to pathological stage, lymph node metastasis, and BMIs ≥25. No significant differences between the two surgical methods were noted, further indicating that the robotic surgical system may have the potential to become a substitute for traditional laparoscopic surgery. With the emergence of new technologies, the application prospects of robotic surgery are promising.

This study had several limitations. First, this was a retrospective study that lacked subjective indicators of postoperative rehabilitation, and further multicenter, large sample, high‐quality RCT studies to confirm these findings are warranted. Second, the long‐term effects of the two groups were comparable; however, the RTG group had better short‐term outcomes and intraoperative performance, more precise lymph node dissection and damage control, reduced blood loss and transfusion, lower stress responses, faster postoperative recoveries, and earlier initiations of adjuvant chemotherapy. Extended observation periods to assess the importance of these measures in improving the prognoses of patients, such as 5‐year OS and RFS, may be necessary. Third, our center had a large volume of operations, and the operators had a high level of experience in laparoscopic and robotic surgery, which may not be applicable or practical to other centers. In view of the short learning curve of robotic surgery,^12^, ^13^, ^14^ it is reasonable to believe that increased training may help surgeons overcome technical difficulties. Fourth, existing studies show that the application of robots in obese patients had significant prospects. Although this study showed extraordinary results, further research involving these patients may be needed, such as our currently ongoing multicenter randomized controlled trial (NCT04636099) in patients with BMI ≥25.0 kg/m^2^ undergoing laparoscopic or robotic gastrectomy (Shandong Gastrointestinal surgery study group, GISSG 20‐01 study).

## 5 CONCLUSION

Although the long‐term outcomes of the two methods were similar, compared with LTG, RTG may improve short‐term clinical outcomes, reduce surgical trauma, reduce surgeon fatigue, reduce inflammatory reactions, and accelerate postoperative recovery. Robotic surgical systems may also reduce the surgical stress response, which may be an underlying mechanism in improving tumor prognosis in gastric cancer patients. For obese or overweight patients with BMIs ≥25, RTG had clear advantages in visual field exposure, surgical outcomes, postoperative recoveries, and lymph node dissections. Robotic surgery systems may be an improved alternative to traditional laparoscopic surgery and may have broader application prospects.

## AUTHOR CONTRIBUTIONS

**Zhuoyu Jia:** Conceptualization (equal); data curation (equal); formal analysis (equal); funding acquisition (equal); methodology (equal); project administration (equal); resources (equal); software (equal); supervision (equal); validation (equal); visualization (equal); writing – original draft (equal); writing – review and editing (equal). **Shougen Cao:** Conceptualization (equal); methodology (equal); project administration (equal); resources (equal); supervision (equal). **Cheng Meng:** Conceptualization (equal); formal analysis (equal); methodology (equal); project administration (equal); resources (equal); software (equal). **Xiaodong Liu:** Data curation (equal); methodology (equal); resources (equal); visualization (equal). **Zequn Li:** Data curation (equal); formal analysis (equal); funding acquisition (equal); resources (equal); supervision (equal); validation (equal). **Yulong Tian:** Data curation (equal); methodology (equal); project administration (equal); resources (equal); software (equal); visualization (equal). **Junjian Yu:** Methodology (equal); project administration (equal); software (equal); supervision (equal); writing – original draft (equal). **Yuqi sun:** Formal analysis (equal); methodology (equal); project administration (equal). **Jianfei Xu:** Data curation (equal); formal analysis (equal); methodology (equal); resources (equal). **Gan Liu:** Data curation (equal); formal analysis (equal); investigation (equal); methodology (equal); project administration (equal). **Xingqi Zhang:** Data curation (equal); methodology (equal); resources (equal). **Hao Yang:** Methodology (equal); project administration (equal); visualization (equal). **Hao Zhong:** Formal analysis (equal); methodology (equal); project administration (equal). **Qingrui Wang:** Methodology (equal); project administration (equal); visualization (equal). **Yanbing Zhou:** Conceptualization (equal); data curation (equal); methodology (equal); project administration (equal).

## FUNDING INFORMATION

This study was funded by Shandong Provincial Natural Science Foundation, China (No. ZR202103040182); the National Natural Science Youth Foundation of China (No. 82103577).

## CONFLICT OF INTEREST STATEMENT

The authors declare that they have no conflict of interest.

## ETHICS STATEMENT

This study was approved by the ethics committee, ethics number: QYFY WZLL 27151.

## Supporting information


Figure S5.
Figure S6.

## Data Availability

Original data are available from the corresponding author, Yanbing Zhou, on request

## References

[1] Sung H , Ferlay J , Siegel RL , et al. Global cancer statistics 2020: Globocan estimates of incidence and mortality worldwide for 36 cancers in 185 countries. CA Cancer J Clin. 2021;71:209‐249. 33538338 10.3322/caac.21660

[2] Kitano S , Iso Y , Moriyama M , Sugimachi K . Laparoscopy‐assisted billroth i gastrectomy. Surg Laparosc Endosc. 1994;4:146‐148. 8180768

[3] Kim W , Kim HH , Han SU , et al. Decreased morbidity of laparoscopic distal gastrectomy compared with open distal gastrectomy for stage i gastric cancer: short‐term outcomes from a multicenter randomized controlled trial (klass‐01). Ann Surg. 2016;263:28‐35. 26352529 10.1097/SLA.0000000000001346

[4] Kinoshita T , Uyama I , Terashima M , et al. Long‐term outcomes of laparoscopic versus open surgery for clinical stage ii/iii gastric cancer: a multicenter cohort study in Japan (loc‐a study). Ann Surg. 2019;269:887‐894. 29697447 10.1097/SLA.0000000000002768

[5] Wong J . Effect of laparoscopic vs. Open distal gastrectomy on 3‐year disease free survival in patients with locally advanced gastric cancer: commentary on the class‐01 randomized clinical trial. Translational Gastroenterology and Hepatology. 2019;4:78. 31872142 10.21037/tgh.2019.09.14 PMC6917552

[6] Nakauchi M , Vos E , Janjigian YY , et al. Comparison of long‐ and short‐term outcomes in 845 open and minimally invasive gastrectomies for gastric cancer in the United States. Ann Surg Oncol. 2021;28:3532‐3544. 33709174 10.1245/s10434-021-09798-y PMC8323986

[7] Jin SH , Kim DY , Kim H , et al. Multidimensional learning curve in laparoscopy‐assisted gastrectomy for early gastric cancer. Surg Endosc. 2007;21:28‐33. 16960676 10.1007/s00464-005-0634-3

[8] Anderson C , Ellenhorn J , Hellan M , Pigazzi A . Pilot series of robot‐assisted laparoscopic subtotal gastrectomy with extended lymphadenectomy for gastric cancer. Surg Endosc. 2007;21:1662‐1666. 17345142 10.1007/s00464-007-9266-0

[9] Song J , Oh SJ , Kang WH , Hyung WJ , Choi SH , Noh SH . Robot‐assisted gastrectomy with lymph node dissection for gastric cancer: lessons learned from an initial 100 consecutive procedures. Ann Surg. 2009;249:927‐932. 19474671 10.1097/01.sla.0000351688.64999.73

[10] Hashizume M , Konishi K , Tsutsumi N , Yamaguchi S , Shimabukuro R . A new era of robotic surgery assisted by a computer‐enhanced surgical system. Surgery. 2002;131:S330‐S333. 11821833 10.1067/msy.2002.120119

[11] Hashizume M , Shimada M , Tomikawa M , et al. Early experiences of endoscopic procedures in general surgery assisted by a computer‐enhanced surgical system. Surg Endosc. 2002;16:1187‐1191. 11984681 10.1007/s004640080154

[12] Strong VE , Russo AE , Nakauchi M , et al. Robotic gastrectomy for gastric adenocarcinoma in the USA: insights and oncologic outcomes in 220 patients. Ann Surg Oncol. 2021;28:742‐750. 32656721 10.1245/s10434-020-08834-7 PMC8323985

[13] Zheng‐Yan L , Feng Q , Yan S , et al. Learning curve of robotic distal and total gastrectomy. Br J Surg. 2021;108:1126‐1132. 34037206 10.1093/bjs/znab152

[14] Kim MS , Kim WJ , Hyung WJ , et al. Comprehensive learning curve of robotic surgery: discovery from a multicenter prospective trial of robotic gastrectomy. Ann Surg. 2021;273:949‐956. 31503017 10.1097/SLA.0000000000003583

[15] Li ZY , Zhou YB , Li TY , et al. Robotic gastrectomy versus laparoscopic gastrectomy for gastric cancer: a multicenter cohort study of 5402 patients in China. Ann Surg. 2021;277:e87‐e95. 34225299 10.1097/SLA.0000000000005046

[16] Choi S , Song JH , Lee S , et al. Trends in clinical outcomes and long‐term survival after robotic gastrectomy for gastric cancer: a single high‐volume center experience of consecutive 2000 patients. Gastric Cancer. 2022;25:275‐286. 34405291 10.1007/s10120-021-01231-3

[17] Choi S , Song JH , Lee S , et al. Surgical merits of open, laparoscopic, and robotic gastrectomy techniques with d2 lymphadenectomy in obese patients with gastric cancer. Ann Surg Oncol. 2021;28:7051‐7060. 33834323 10.1245/s10434-021-09952-6

[18] Tian Y , Cao S , Kong Y , et al. Short‐ and long‐term comparison of robotic and laparoscopic gastrectomy for gastric cancer by the same surgical team: a propensity score matching analysis. Surg Endosc. 2022;36:185‐195. 33427913 10.1007/s00464-020-08253-5

[19] Ojima T , Nakamura M , Hayata K , et al. Short‐term outcomes of robotic gastrectomy vs laparoscopic gastrectomy for patients with gastric cancer: a randomized clinical trial. JAMA Surg. 2021;156:954‐963. 34468701 10.1001/jamasurg.2021.3182 PMC8411361

[20] Chen QY , Zhong Q , Liu ZY , et al. Surgical outcomes, technical performance and surgery burden of robotic total gastrectomy for locally advanced gastric cancer: a prospective study. Ann Surg. 2021;276:e434‐e443. 33491975 10.1097/SLA.0000000000004764

[21] Li Z , Qian F , Zhao Y , et al. A comparative study on perioperative outcomes between robotic versus laparoscopic d2 total gastrectomy. Int J Surg. 2022;102:106636. 35472517 10.1016/j.ijsu.2022.106636

[22] Yang C , Shi Y , Xie S , et al. Short‐term outcomes of robotic‐ versus laparoscopic‐assisted total gastrectomy for advanced gastric cancer: a propensity score matching study. BMC Cancer. 2020;20:669. 32680479 10.1186/s12885-020-07160-1 PMC7367399

[23] Japanese gastric cancer treatment guidelines . 5th edition. Gastric Cancer. 2018;2021(24):1‐21. 10.1007/s10120-020-01042-y PMC7790804 32060757

[24] Liu F , Huang C , Xu Z , et al. Morbidity and mortality of laparoscopic vs open total gastrectomy for clinical stage i gastric cancer: the class02 multicenter randomized clinical trial. JAMA Oncol. 2020;6:1590‐1597. 32815991 10.1001/jamaoncol.2020.3152 PMC7441466

[25] Marano L , Fusario D , Savelli V , Marrelli D , Roviello F . Robotic versus laparoscopic gastrectomy for gastric cancer: An umbrella review of systematic reviews and meta‐analyses. Updates Surg. 2021;73:1673‐1689. 34031848 10.1007/s13304-021-01059-7 PMC8500879

[26] Marano L , D'Ignazio A , Resca L , Marrelli D , Roviello F . Robotic‐assisted gastrectomy for gastric cancer: single western center results. Updates Surg. 2021;73:865‐872. 33058054 10.1007/s13304-020-00896-2 PMC8184723

[27] Marano L , Fusario D , Savelli V , et al. Robotic versus laparoscopic gastrectomy for gastric cancer: protocol for umbrella review of systematic reviews and meta‐analyses. BMJ Open. 2020;10:e033634. 10.1136/bmjopen-2019-033634 PMC7050371 32111613

[28] Amin MB , Greene FL , Edge SB , et al. The eighth edition ajcc cancer staging manual: continuing to build a bridge from a population‐based to a more "personalized" approach to cancer staging. CA Cancer J Clin. 2017;67:93‐99. 28094848 10.3322/caac.21388

[29] Kang BH , Xuan Y , Hur H , Ahn CW , Cho YK , Han SU . Comparison of surgical outcomes between robotic and laparoscopic gastrectomy for gastric cancer: the learning curve of robotic surgery. J Gastric Cancer. 2012;12:156‐163. 23094227 10.5230/jgc.2012.12.3.156 PMC3473222

[30] Tian Y , Cao S , Li L , et al. Effects of perioperative enhanced recovery after surgery pathway management versus traditional management on the clinical outcomes of laparoscopic‐assisted radical resection of distal gastric cancer: study protocol for a randomized controlled trial. Trials. 2020;21:369. 32357913 10.1186/s13063-020-04272-8 PMC7193340

[31] Kehlet H . Multimodal approach to control postoperative pathophysiology and rehabilitation. Br J Anaesth. 1997;78:606‐617. 9175983 10.1093/bja/78.5.606

[32] Wang D , Kong Y , Zhong B , Zhou X , Zhou Y . Fast‐track surgery improves postoperative recovery in patients with gastric cancer: a randomized comparison with conventional postoperative care. J Gastrointest Surg. 2010;14:620‐627. 20108171 10.1007/s11605-009-1139-5

[33] Sugisawa N , Tokunaga M , Makuuchi R , et al. A phase ii study of an enhanced recovery after surgery protocol in gastric cancer surgery. Gastric Cancer. 2016;19:961‐967. 26260875 10.1007/s10120-015-0528-6

[34] Shen S , Cao S , Jiang H , et al. The short‐term outcomes of gastric cancer patients based on a proposal for a novel classification of perigastric arteries. J Gastrointest Surg. 2020;24:2471‐2481. 31749096 10.1007/s11605-019-04427-2

[35] Inaba K , Satoh S , Ishida Y , et al. Overlap method: novel intracorporeal esophagojejunostomy after laparoscopic total gastrectomy. J Am Coll Surg. 2010;211:e25‐e29. 21036074 10.1016/j.jamcollsurg.2010.09.005

[36] Huang CM , Huang ZN , Zheng CH , et al. An isoperistaltic jejunum‐later‐cut overlap method for esophagojejunostomy anastomosis after totally laparoscopic total gastrectomy: a safe and feasible technique. Ann Surg Oncol. 2017;24:1019‐1020. 27921193 10.1245/s10434-016-5658-5

[37] Kwon IG , Son YG , Ryu SW . Novel intracorporeal esophagojejunostomy using linear staplers during laparoscopic total gastrectomy: Π‐shaped esophagojejunostomy, 3‐in‐1 technique. J Am Coll Surg. 2016;223:e25‐e29. 27370184 10.1016/j.jamcollsurg.2016.06.011

[38] Lobo DN , Gianotti L , Adiamah A , et al. Perioperative nutrition: recommendations from the espen expert group. Clinical Nutrition (Edinburgh, Scotland). 2020;39:3211‐3227. 32362485 10.1016/j.clnu.2020.03.038

[39] Dindo D , Demartines N , Clavien PA . Classification of surgical complications: a new proposal with evaluation in a cohort of 6336 patients and results of a survey. Ann Surg. 2004;240:205‐213. 15273542 10.1097/01.sla.0000133083.54934.ae PMC1360123

[40] Katayama H , Kurokawa Y , Nakamura K , et al. Extended clavien‐dindo classification of surgical complications: Japan clinical oncology group postoperative complications criteria. Surg Today. 2016;46:668‐685. 26289837 10.1007/s00595-015-1236-x PMC4848327

[41] Hikage M , Fujiya K , Kamiya S , Tanizawa Y , Bando E , Terashima M . Comparisons of surgical outcomes between robotic and laparoscopic total gastrectomy in patients with clinical stage i/iia gastric cancer. Surg Endosc. 2022;36:5257‐5266. 34997341 10.1007/s00464-021-08903-2

[42] Roh CK , Lee S , Son SY , Hur H , Han SU . Textbook outcome and survival of robotic versus laparoscopic total gastrectomy for gastric cancer: a propensity score matched cohort study. Sci Rep. 2021;11:15394. 34321568 10.1038/s41598-021-95017-3 PMC8319437

[43] Smith DD , Schwarz RR , Schwarz RE . Impact of total lymph node count on staging and survival after gastrectomy for gastric cancer: data from a large us‐population database. J Clin Oncol. 2005;23:7114‐7124. 16192595 10.1200/JCO.2005.14.621

[44] Jiao XG , Deng JY , Zhang RP , et al. Prognostic value of number of examined lymph nodes in patients with node‐negative gastric cancer. World J Gastroenterol. 2014;20:3640‐3648. 24707149 10.3748/wjg.v20.i13.3640 PMC3974533

[45] Son T , Hyung WJ , Lee JH , et al. Clinical implication of an insufficient number of examined lymph nodes after curative resection for gastric cancer. Cancer. 2012;118:4687‐4693. 22415925 10.1002/cncr.27426

[46] Wu CW , Hsiung CA , Lo SS , et al. Nodal dissection for patients with gastric cancer: a randomised controlled trial. Lancet Oncol. 2006;7:309‐315. 16574546 10.1016/S1470-2045(06)70623-4

[47] Degiuli M , Sasako M , Ponti A , et al. Randomized clinical trial comparing survival after d1 or d2 gastrectomy for gastric cancer. Br J Surg. 2014;101:23‐31. 24375296 10.1002/bjs.9345

[48] Okabe H , Obama K , Tsunoda S , et al. Feasibility of robotic radical gastrectomy using a monopolar device for gastric cancer. Surg Today. 2019;49:820‐827. 30929081 10.1007/s00595-019-01802-z

[49] de Leval MR , Carthey J , Wright DJ , Farewell VT , Reason JT . Human factors and cardiac surgery: a multicenter study. J Thorac Cardiovasc Surg. 2000;119:661‐672. 10733754 10.1016/S0022-5223(00)70006-7

[50] Fecso AB , Bhatti JA , Stotland PK , Quereshy FA , Grantcharov TP . Technical performance as a predictor of clinical outcomes in laparoscopic gastric cancer surgery. Ann Surg. 2019;270:115‐120. 29578907 10.1097/SLA.0000000000002741

[51] Lu J , Zheng CH , Xu BB , et al. Assessment of robotic versus laparoscopic distal gastrectomy for gastric cancer: a randomized controlled trial. Ann Surg. 2021;273:858‐867. 32889876 10.1097/SLA.0000000000004466

[52] Shibasaki S , Nakauchi M , Serizawa A , et al. Clinical advantage of standardized robotic total gastrectomy for gastric cancer: a single‐center retrospective cohort study using propensity‐score matching analysis. Gastric Cancer. 2022;25:804‐816. 35298742 10.1007/s10120-022-01288-8

[53] Suda K , Yamamoto H , Nishigori T , et al. Safe implementation of robotic gastrectomy for gastric cancer under the requirements for universal health insurance coverage: a retrospective cohort study using a nationwide registry database in Japan. Gastric Cancer. 2022;25:438‐449. 34637042 10.1007/s10120-021-01257-7 PMC8505217

