# Supplementary material for: Intraoperative performance and outcomes of robotic and laparoscopic total gastrectomy for gastric cancer: A high‐volume center retrospective propensity score matching study
Source: Cancer Med. 2023 Mar 16;12(9):10485–98. doi: 10.1002/cam4.5785 (PMC10225175; doi:10.1002/cam4.5785)
Supplement: Supplementary file 1 — Figure S5. Figure S6. [file CAM4-12-10485-s001.docx]

**eFigure**

**eFig.5.** Kaplan–Meier estimates of relapse-free survival of all patients (**A**), patients with stage I disease (**B**), patients with stage II disease (**C**), and patients with stage III disease (**D**)

**eFig.6.** Kaplan–Meier estimates of survival for status lymph nodes, all N0 patients OS (**A**), all N0 patients RFS (**B**), all N+ patients OS (**C**), all N+ patients RFS (**D**), all BMIs ≥ 25 patients OS (**E**), and all BMIs ≥ 25 patients RFS (**F**)

**eFig.5**


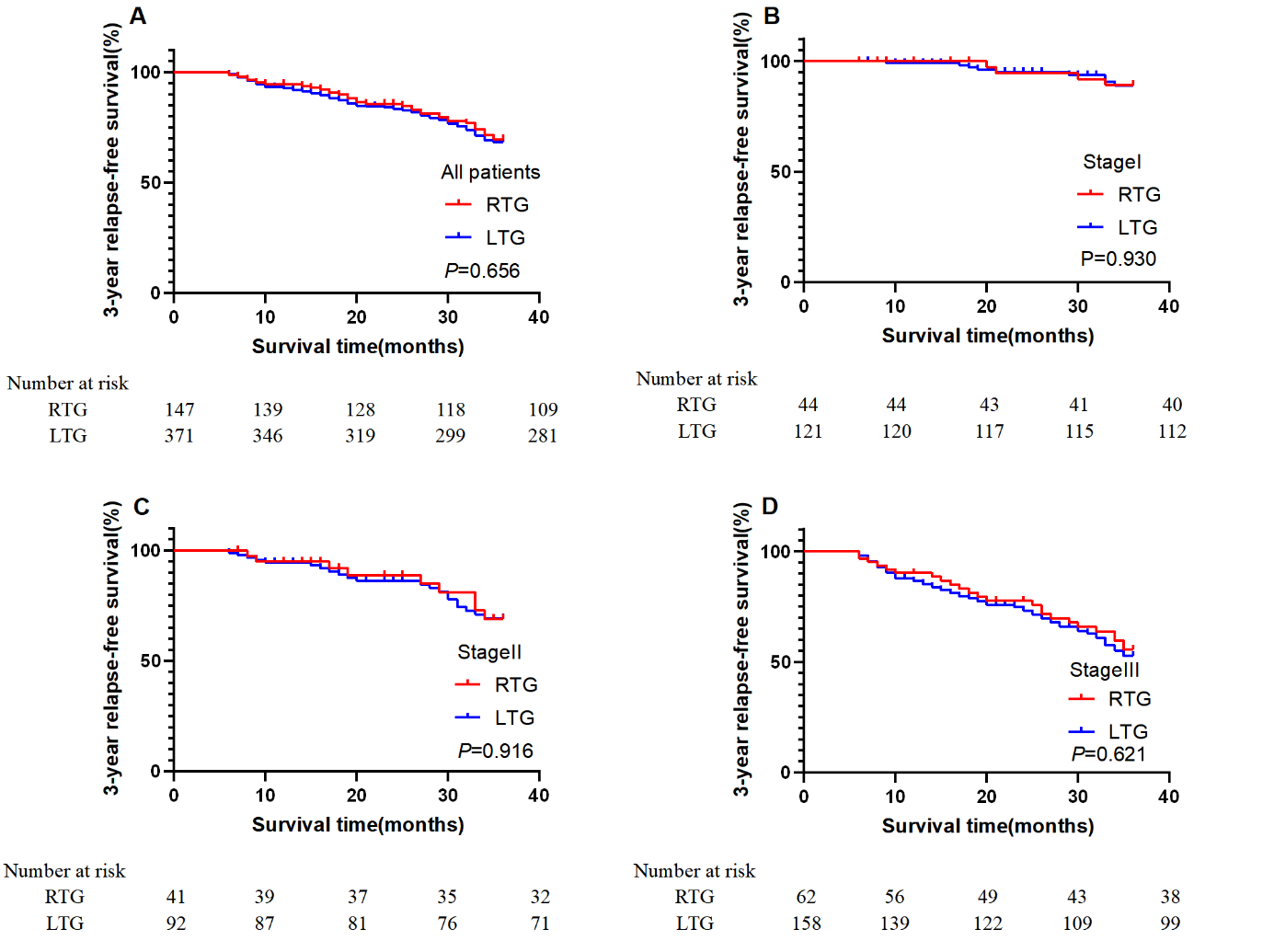


**eFig.****6**

**
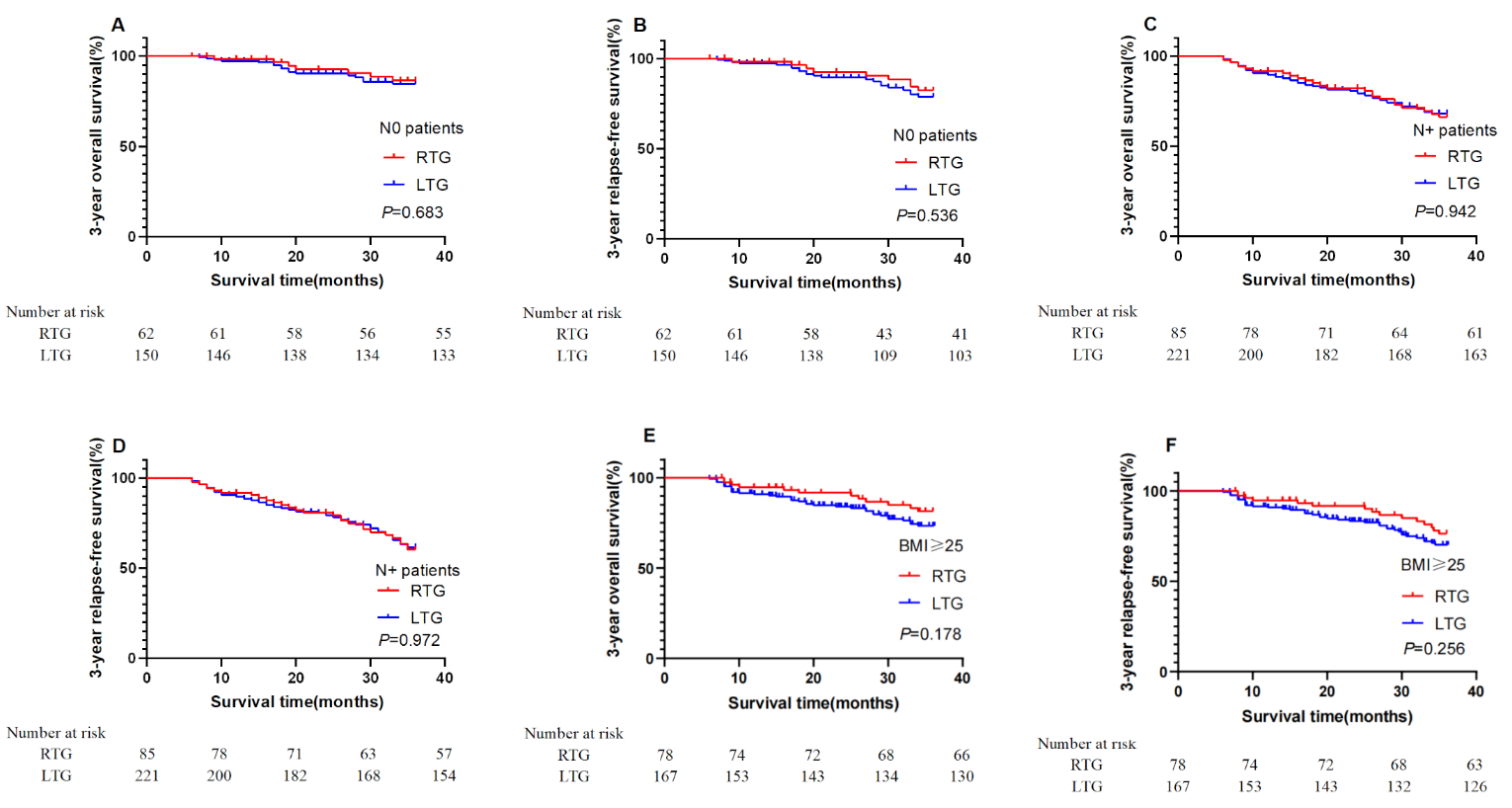
**
